# Evolutionary Diversification of the Lizard Genus *Bassiana* (Scincidae) across Southern Australia

**DOI:** 10.1371/journal.pone.0012982

**Published:** 2010-09-24

**Authors:** Sylvain Dubey, Richard Shine

**Affiliations:** School of Biological Sciences, University of Sydney, Sydney, New South Wales, Australia; Natural History Museum of Denmark, Denmark

## Abstract

**Background:**

Relatively recent (Plio-Pleistocene) climatic variations had strong impacts on the fauna and flora of temperate-zone North America and Europe; genetic analyses suggest that many lineages were restricted to unglaciated refuges during this time, and have expanded their ranges since then. Temperate-zone Australia experienced less severe glaciation, suggesting that patterns of genetic structure among species may reflect older (aridity-driven) divergence events rather than Plio-Pleistocene (thermally-mediated) divergences. The lizard genus *Bassiana* (Squamata, Scincidae) contains three species that occur across a wide area of southern Australia (including Tasmania), rendering them ideally-suited to studies on the impact of past climatic fluctuations.

**Methodology/Principal Findings:**

We performed molecular phylogenetic and dating analyses using two partial mitochondrial genes (*ND2* and *ND4*) of 97 samples of *Bassiana* spp. Our results reveal a pattern of diversification beginning in the Middle Miocene, with intraspecific diversification arising from 5.7 to 1.7 million years ago in the Upper Miocene-Lower Pleistocene.

**Conclusions/Significance:**

In contrast to the temperate-zone Northern Hemisphere biota, patterns of evolutionary diversification within southern Australian taxa appear to reflect geologically ancient events, mostly relating to east-west discontinuities imposed by aridity rather than (as is the case in Europe and North America) relatively recent recolonisation of northern regions from unglaciated refugia to the south.

## Introduction

The geographic distribution of genetic diversity within a biological lineage can tell us a great deal about the processes that have affected those organisms over evolutionary time. For example, major genetic discontinuities can reveal places where barriers to dispersal occurred, often reflecting areas unsuitable for the species' persistence at some time in the past [1]. Information on the timing of such events, also obtainable from genetic data, can help to identify the nature of processes (e.g., climatic variation, sea-level fluctuation, volcanic activity) that created barriers to gene flow.

Palynological data suggest that the impact of climatic variation on the vegetation was weaker in the Southern Hemisphere than in the North, because large ice sheets were rare and full glacial conditions did not persist during interglacial periods; thus, forested or partially forested areas existed in mesic habitats of southeastern and southwestern Australia even during the Last Glacial Maximum [2]. Nonetheless, the distributions of particular vegetation types presumably expanded and contracted during the glacial fluctuations of the Upper Pliocene and Pleistocene [3], [4]. Also, extreme arid conditions rendered large regions of inland Australia unsuitable for vegetation, leading to the formation of active sand and clay dune systems. Clearly, these modifications would have had a substantial impact on the distributions of all types of fauna in arid temperate areas [5].

Although the impacts of Pleistocene climatic variations on the fauna of southern Australia remain unclear, the past climatic history of southern Australia is relatively well-understood. From the Lower-Middle Miocene (23 mya [million years ago]), Australia was warm and wet, even in areas now covered by desert [6]. Further south in Antarctica, ice was absent until 14 mya [7]. In the Upper Miocene, the disappearance of these stable, warm, wet conditions caused a contraction of rainforest, an expansion of xerophyllous plant species such as *Eucalyptus* and Casuarinaceae, and the retreat of the southern marine basin (e.g., the Murray and Gippsland basins in southeastern Australia: [5], [8]. Although warm and wet conditions (and thus, rainforest) returned in the Lower Pliocene (5−3 mya), climatic fluctuations (cool-dry to warm-wet) and a global cooling-drying trend through the Pliocene favoured the expansion of woodlands, sclerophyllous forest and grasslands (but still wetter than today; [5], [8]–[12]). The beginning of the Pleistocene marked a transition to cold and dry conditions, and the lower sea level (to more than 120 m lower; [13], [14]) periodically connected continental islands to the mainland (e.g. Tasmania; [15]). Pleistocene climatic fluctuations reached a maximum amplitude over the last 0.4 Myr, with temperatures 10 to 5°C lower during the last Glacial Maximum 21,000 years ago than present-day conditions and/or fully arid conditions (LGM; [5], [8], [16]). However, glaciation was limited to Tasmania and the southeastern highlands [8].

**Figure 1:**
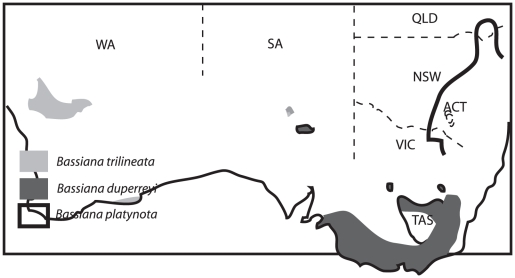
Distribution of *Bassiana* species in southern Australia.

These climatic fluctuations were likely to exert stronger effects on the distribution and abundance of some species than others. For example, terrestrial ectotherms (such as reptiles) depend upon ambient thermal heterogeneity for behavioural thermoregulation, and hence are sensitive to local climatic conditions (e.g. [17], [18]). Consequently, reptiles provide excellent model systems with which to study the impact of past climatic variations, because the genetic signatures of past events are likely to be discernable in these taxa. In the present study, we performed molecular phylogenetic and dating analyses on a widespread genus of lizards (*Bassiana*, Scincidae, Squamata) from southern Australia (Fig. 1), to document patterns of genetic diversification within this group and to clarify potential impacts of past climatic variations and geographic barriers on gene flow in this taxon.

## Results

### Phylogenetics analyses

**Figure 2:**
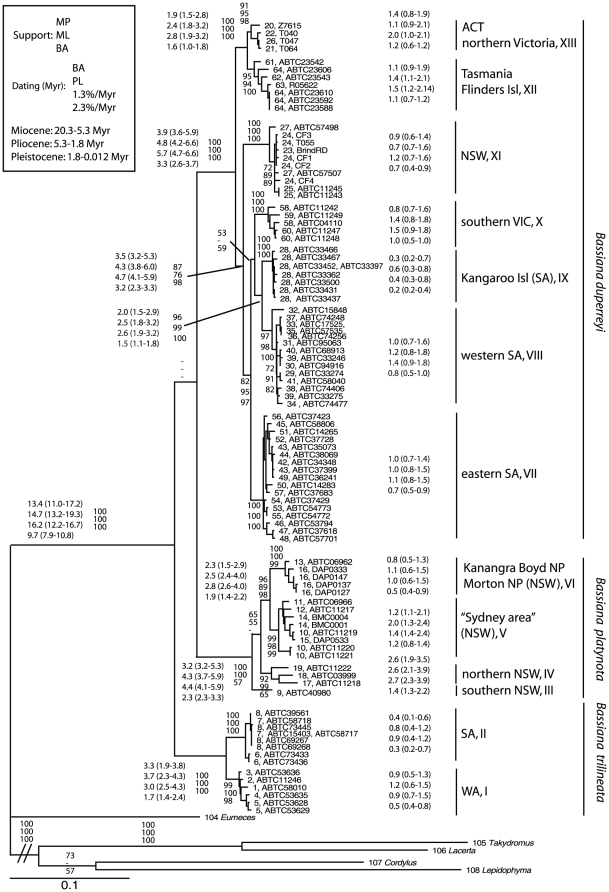
Phylogeny of the 1412 bp *ND2*+*ND4* fragment of the scincid lizard genus *Bassiana* in southern Australia analysed using a maximum likelihood (ML) procedure and the TrN+I+G model of substitution. Support values shown for the major clades only for maximum parsimony (MP), maximum likelihood (ML), and Bayesian (BA) analyses; and dating of the major splits in Myr from the Beast analyses, with the secondary calibrations points from Albert et al. (2009) respectively BA-based and PL-based, and the divergence rates of respectively 1.3%/Myr and 2.3%/Myr. Codes are as in Table S1.

The 97 samples showed 92 different haplotypes of 1412 bp (combined dataset; GenBank accession numbers GU811882– GU812071), containing 414 variable sites, of which 353 were parsimony-informative (excluding outgroups). Because trees from the BA, ML and MP analyses showed very similar relationships, only the ML tree is shown (with ML, MP, and BA support values: Fig. 2).

The genus *Bassiana*, as well as the three species within it, formed monophyletic units (support of 100, 100 and 1.0 for MP, ML, and BA analyses except for *Bassiana platynota* where BA analyses provided only poorly support for its monophyly; Fig. 2). However, the phylogenetic relationships between the three species remain unclear.

**Figure 3:**
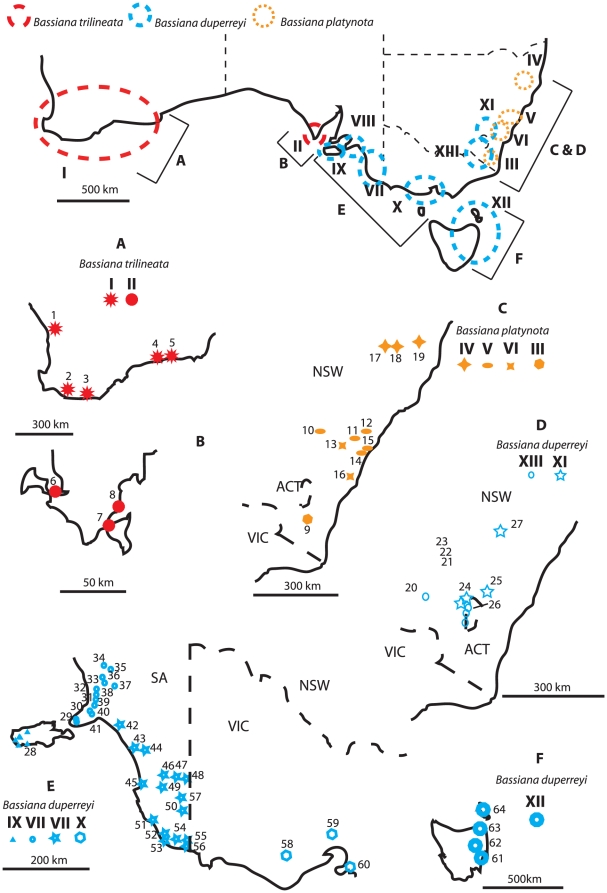
Distribution of *Bassiana* samples and genetic lineages from the present study (A–B: *B. trilineata*; C, E, and F: *B. duperreyi*; D: *B. platynota*). Codes are as in Table S1.

Major phylogenetic divisions were evident within each of the three *Bassiana* species. Two well-supported lineages corresponding to different geographic locations were found within *B. trilineata* (I: Western Australia; II: South Australia, Figs. 2 and 3), seven geographically localised lineages within *B. duperreyi* (IX: Kangaroo Island, VIII: western SA, V: eastern SA, X: southern VIC, XII: TAS, XIII: northern VIC and ACT, XI: NSW), and four within *B. platynota* (IV: northern NSW, V: Sydney area, VI: Kanangra Boyd and Morton NP; III: southern NSW).

The three species are well-separated genetically. Mean K2P genetic distances between species were 11.1% for *duperreyi*-*platynota*, 12.3% for *duperreyi*-*trilineata*, and 12.8% for *platynota*-*trilineata*. The genetic distance between the two lineages of *B. trilineata* was 2.9%, whereas the genetic distances between the lineages of *B. duperreyi* varied from 2.3 (XII-XIII) to 5.3% (VIII-XII), and between the lineages of *B. platynota* from 3.3 (V–VI) to 4.7% (III–IV). Genetic distances within lineages varied from 0.3 (IV) to 1.1% (XII) in *B. duperreyi*, from 0.8 (VI) to 3.1% (IV; excluding lineage III represented by one sample) in *B. platynota*, and from 0.5 (II) to 0.8% (I) in *B. trilineata*.

### Dating analyses

The phylogenetic split between the three *Bassiana* species occurred in the Miocene (BA: 13.4 Myr, 95% HPD: 11.0–17.2; PL: 14.7, 95% HPD 13.2–19.3; 1.3%: 16.2 Myr, 95% HPD: 12.2–16.7; 2.3%: 9.7 Myr, 95% HPD: 7.9–10.8; datings of divergence points are given in the same order below; see Fig. 3 for more details). Within *B. duperreyi*, the estimates dates of divergence between the major lineages began in the Upper Miocene and/or Upper Pliocene (3.9 Myr, 95% HPD: 3.6–5.9; 4.8, 95% HPD 4.2–6.6; 5.7 Myr, 95% HPD: 4.7–6.6; 3.3 Myr, 95% HPD: 2.6–3.7), depending on the calibration method. Within *B. platynota*, the splits between the major lineages began in the Lower and Upper Pliocene (3.2 Myr, 95% HPD: 3.2–5.3; 4.3, 95% HPD 3.7–5.9; 4.4 Myr, 95% HPD: 4.1–5.9; 2.3 Myr, 95% HPD: 2.3–3.3), and within *B. trilineata* from the Lower Pliocene to the Lower Pleistocene between the two main lineages (3.3 Myr, 95% HPD: 1.9–3.8; 3.7, 95% HPD 2.3–4.3; 3.0 Myr, 95% HPD: 2.5–4.3; 1.7 Myr, 95% HPD: 1.4–2.4), again depending on the calibration method. Finally, diversification within each of these intraspecific lineages occurred from the Upper Miocene in lineage VIII of *B. duperreyi* (based on a divergence rate of 1.3% Myr^−1^; 2.0 Myr 95% HPD: 1.0–2.1) to the Middle Pleistocene in lineage III of *B. duperreyi* (based on a divergence rate of 2.3% Myr^−1^; 0.2 Myr 95% HPD: 0.2–0.4; see Fig. 3 for more details about the dating results).

These data suggest that five distinct lineages of *B. duperreyi* occur in mainland Australia, plus one in Tasmania and Flinders Island, and one in Kangaroo Island (SA). An early split (Upper Miocene-Upper Pliocene) occurred between lineages in south-eastern Australia (XII: TAS; XIII: ACT, Northern VIC, bioregion of South Eastern Highland and Australian Alps) and the other (northern and western) conspecifics, followed by a second split between eastern (XI: NSW, South Eastern Highland) and western populations (IX to X: VIC and SA) in the Upper-Lower Pliocene. The lineages of *B. duperreyi* from SA and southern VIC occur in different bioregions, respectively in the Murray Darling Depression and Kanmantoo region (VIII), Naracoorte Coastal Plain (VII), Victorian Volcanic Plain and South East Coastal Plain (X; [19]), and appear to have diversified in the Upper Pliocene-Lower Pleistocene.

## Discussion

Our analyses reveal strong biogeographic structure within *Bassiana* in southern Australia, with an initial diversification of the genus occurring in the Miocene (16.2−9.7 mya; fig. 2), at about the same time that climatic conditions in southern Australia became less stable and more arid [5]. Diversification within each of the three *Bassiana* species also began long ago, probably from the Upper Miocene (5.7 Myr) to the Lower Pleistocene (1.6 Myr). Most of this intraspecific diversification occurred during the Pliocene (Fig. 2), a period marked by a global cooling and drying trend [9]–[11]. Plausibly, those climatic changes rendered many parts of Australia unsuitable for these lizards, splitting formerly continuous populations into at least 13 major genetic lineages, each in a different geographic region.

Although mainland Australia and Tasmania currently are separated by Bass Strait (average depth of 60 m: [20]), variations in sea level created land bridges (and thus, opportunities for terrestrial dispersal of lizards) several times over the last 10 Myr. At least two such events occurred during the last 0.5 Myr, with sea levels up to 150 m lower than at present [15]. Similar connections would have occurred between Kangaroo Island and mainland (currently separated by the Backstairs Passage with an average depth of 36 m). The split between insular populations of *B. duperreyi* in Tasmania (XII) and Kangaroo Island (IX) and their most closely related mainland populations, during the Upper Pliocene-Lower Pleistocene is in accord with these sea-level-driven dispersal opportunities. Similarly, the current east-west split in *B. trilineata* populations (WA vs SA's Eyre Peninsula) reflects the inhospitable habitats of the arid Nullarbor Plain, and hence a cessation of genetic exchange during the Lower Pliocene and Upper Pleistocene. In *B. platynota*, the same pattern is observed: diversification beginning in the Lower and/or Upper Pliocene and dividing populations into four main lineages, one in the South Eastern Highlands (III), two in the Sydney Basin (South: VI and North: V), and one on the NSW North Coast (IV).

The considerable antiquity of these phylogenetic divergences within *Bassiana* also is evident from the diversification of haplotypes within intraspecific lineages (except lineage XIII of *B. duperreyi*, represented by only one sample). Many of these divergences appear to date back to the Upper Pliocene through to the Lower and Middle Pleistocene. Any impact of the last Glacial Maximum (21,000 years ago; [16]) would be apparent in genetic homogeneity over large areas (reflecting recent recolonisation) and/or the elimination of the genetic signatures of more ancient divergence events (because of extirpation of populations over wide areas). Neither of these patterns is apparent, suggesting that these recent climatic fluctuations had no substantial impact on present genetic diversity within lineages. Conditions over most of southern Australia appear to have remained suitable for population persistence, allowing endemic lineages of these lizards to persist.

In summary, lizards of the genus *Bassiana* have evolved in southern Australia over a period of at least 10 million years (interspecific divergences), and most of the major intraspecific phylogenetic divergences among this lineage occurred at least 5.7 million years ago to 1.7 million years ago. Climatic fluctuations during the Upper Pleistocene (including the Last Glacial Maximum) do not appear to have substantially affected the extent of areas within lizards could persist, with the result that the genetic signature of ancient divergence events (between and within lineages) remains clearly expressed in modern-day populations in the form of strong geographically-associated genetic structure.

These conclusions accord well with the results of phylogenetic analyses of other reptile and amphibian lineages within southern Australia. Some widespread taxa show little geographic genetic structure, presumably reflecting recolonisation events after older populations were wiped out by relatively recent (Pleistocene) sea-level fluctuations (tigersnake *Notechis scutatus*; [21]). More commonly, however, reptile and amphibian lineages in southern Australia are highly structured genetically, and reflect the results of ancient diversifications. For example, geckos of the *Diplodactylus vittatus* complex began to diverge about 20–25 mya, in the Lower Miocene and Pliocene [22]). Unsurprisingly, different lineages have reacted to historical climatic fluctuations in different ways. For example, a deep divergence between mainland Australia and Tasmania is evident in some lineages (e.g., the skinks *Lerista bougainvilli* [23] and *Bassiana duperreyi* [current study], and the froglet *Crinia signifera* [24]) whereas animals in the two areas are similar genetically in other taxa (e.g., the frogs *Limnodynastes peronii* and *L. tasmaniensis* [25], the skink *Egernia whitii* [26], and the snake *Notechis scutatus* [21]). Plausibly, such interspecific differences in spatial genetic structure reflect interspecific differences in factors such as habitat requirements, past distributions and dispersal capacities.

Within south and southeastern Australia complex patterns emerge. These include the presence of multiple lineages, as in *Bassiana* species (*duperreyi*, *platynota*, and *trilineata*; this study), *Egernia whitii* [26], *Lerista bougainvilli* [23], *Diplodactylus* [22], and *Crinia signifera* [24]. The skinks *Egernia whitii* [26] and *Bassiana duperreyi* (current study), as well as the frogs *Limnodynastes* spp [25], and *Crinia signifera* [24] show a major divergence between western/eastern or southern/northern populations with a geographic limit situated in Victoria-southern NSW. In *Egernia whitii* and *Crinia signifera*, the geographic breaks among the lineages are consistent with Miocene–Pliocene uplift in the Great Dividing Range, as well as elevated sea levels in East Gippsland [24], [26]. Schaüble & Moritz [25] estimated the split between *Limnodynastes* spp. populations of these two regions to have occurred during the same period.

Similarly, in NSW, major breaks occur between southern and northern populations in taxa such as *Egernia whitii* [26], the *Litoria citropa group* [27], *Oedura lesueurii* [28], *Tympanocryptis* spp [4], *Bassiana platynota* (current study), *Crinia signifera* [24], and *Limnodynastes* spp. [25] with a plausible Miocene and Pliocene diversification in the five latter taxa.

These consistently strong patterns of genetic structure suggest that much of the modern reptile and amphibian fauna of southern Australia bears a strong stamp of divergence events that occurred many million of years ago, and were caused by climatic events that long preceded Pleistocene climatic fluctuations (i.e. in the Miocene and Pliocene). Phylogenetic studies on arid adapted-species from central Australia also suggest a pattern of diversification dating from the mid-Miocene, driven by increasing aridity at that time rather than by thermal fluctuations during the Pleistocene (see [24] for a detailed review). This pattern offers a striking contrast with studies on Northern Hemisphere taxa, where thermal fluctuations (glacial periods) during the Pleistocene eliminated animals from extensive areas, erasing the genetic signature of earlier endemic radiations (e.g. [29]–[32]). Consistent with these studies, a meta-analysis [33] revealed that species of reptiles and amphibians from temperate-zone areas of the Northern Hemisphere tend to be younger (in term of the age of earliest intraspecific diversification) than are taxa found at similar latitudes in the Southern Hemisphere. This long-retained genetic evidence of ancient aridity-driven phylogenetic divergence within presently wide-ranging ectotherms in southern Australia thus provides strong support for the hypothesis that Pleistocene climatic fluctuations in southern Australia were not sufficiently intense to eradicate reptiles and amphibians from most areas.

## Materials and Methods

### Tissue sampling and DNA extraction

The genus *Bassiana* ( =  *Acritoscincus, Eulepis*, or *Leiolopisma* in other taxonomic schemes: e.g., [34]–[36] comprises three recognised species of diurnal ground-dwelling oviparous skinks, all of medium size [37]. The south-western cool-skink *Bassiana trilineata* (Gray, 1838) is distributed in Western Australia (WA) and South Australia (SA), the bold-striped cool-skink *Bassiana duperreyi* (Gray, 1838) in New South Wales (NSW), Victoria (VIC), Tasmania (TAS) and SA (including Kangaroo Island), and the red-throated cool-skink *Bassiana platynota* (Peters, 1881) in NSW and VIC (Fig. 1).

We analysed a total of 97 samples of *Bassiana* from Australian museum collections (Museum Victoria, Australian National Wildlife Collection CSIRO, and South Australian Museum) or collected recently in the field. The study was approved by the University of Sydney Animal Care and Ethics Committee (L04/7-2007/3/4665), and samples were collected in ACT and NSW with the permits LT2009355 and S10826, respectively (see Table S1 for more details). Small tail clips or liver (for samples from museum collections) were used to isolate total cellular DNA, and tissues were placed in 200 µL of 5% Chelex containing 0.2 mg/mL of proteinase K, incubated overnight at 56°C, boiled at 100°C for 10 min, and centrifuged at 13,300 g for 10 min. The supernatant, containing purified DNA, was removed and stored at -20°C.

### DNA amplification

Double-stranded DNA amplifications of NADH dehydrogenase 4 (*ND4*) and 2 (*ND2*) were performed respectively with the primer pairs ND4/LEU [38] and AT4482 (5′caacatgacaaaaattrgcccc3′)/tRNA-ASN (Keogh et al., unpublished; [39]. Amplification conditions included a hot start denaturation of 95°C for 3 min, followed by 35 cycles of 95°C for 60 s, 60°C annealing temperature for 60 s, 72°C for 105 s (*ND2*: 120 s), and a final extension of 72°C for 7 min. Sequence reactions were visualized on a 3730 xl DNA Analyzer (Applied Biosystems, CA, USA).

### Phylogenetic analyses

We aligned sequences using BioEdit [40] and by eye. Concordance of the two genes (*ND2* and *ND4*) used to construct the dataset was evaluated using the partition-homogeneity test with the software PAUP* [41]. As these tests did not reveal significance incongruence between the two genes (*P* = 0.20), we conducted phylogenetic analyses on concatenated sequences (593 bp for *ND2*+819 bp for *ND4*); all codon positions were used. For the combined data set, sequences of species from the infraorder Scincomorpha were used to root the trees, as follows: one species of Cordylidae (*Cordylus warreni*, AB079613), one of Xantusiidae (*Lepidophyma flavimaculatum*, AB162908), two of Lacertidae (*Lacerta viridis*, AM176577; *Takydromus takydromoides*, AB080237), and one of Scincidae (*Eumeces egregius*, AB016606) from Albert et al. [42] and as in Dubey & Shine [43]. All the non-*Bassiana* species were used as outgroups.

For Maximum likelihood (ML) analyses, jModelTest 0.1.1 [44], [45] was used to select models of DNA substitution. The TrN+I+G model [46] best fitted the dataset using a Bayesian Information Criterion (BIC; [47] Schwarz 1978; Lset base = (0.3559 0.3272 0.0877 0.2292) nst = 6 rmat = (1.0000 13.9692 1.0000 1.0000 7.6755 1.0000) rates = gamma shape = 0.4430 pinvar = 0.1490). ML heuristic searches and bootstrap analyses (1000 replicates) were performed with phyml [44]. Bayesian analyses (BA) were conducted with the GTR model (nst = 6), using MrBayes ver. 3.1.2.1 [48]. Two independent runs were performed, each consisting of four parallel Markov chain Monte Carlo (MCMC) chains of 3 million generations, allowing a good convergence of the independent runs (the average standard deviation of split frequencies being lower than 0.01). Trees were sampled every 100 generations. Burn-in was assessed by comparing the mean and variance of log likelihoods, both using the program Tracer ver. 1.4 [49] and by eye. Tree parameters reached stationarity after a burn-in period of 300,000 generations. Optimal trees were then sampled every 100 generations to obtain the final consensus BA tree and associated posterior probabilities.

Finally, we used Paup* 4.0b10 [40] to perform maximum parsimony (MP) analyses using 100 random additions of sequences followed by tree bisection and reconnection (TBR) branch-swapping, and retaining at most 100 trees at each replicate. Branch support was estimated using 1000 bootstrap replicates with the same heuristic settings.

### Molecular dating

We estimated divergence times with Beast 1.4 [50] using a coalescent tree prior (adequate to study intraspecific diversification: [51]. Because the fossil record for Australian skinks is limited [52], we used the combination of three different secondary calibration points from the study by Albert et al. [42] on the phylogeny of squamate reptiles based on two different molecular dating methods (BA: Bayesian, PL: penalized likelihood) and as in the recent study of Dubey & Shine [43] on water skinks (see this study for details of methods and calibration points). Additional simulations were run with the same dataset and the same models, but strictly based on two different rates of divergence (1.3% and 2.3% Myr^−1^) derived from other reliable studies on reptiles [53]–[56].

Preliminary analyses were performed with an uncorrelated lognormal relaxed clock to test if a strict molecular clock can be rejected for our dataset (ucld.stdev parameter >1 with a frequency histogram not abutting 0). Because in our simulations (BA and PL based calibration, and the two divergence rates 1.3% and 2.3% Myr^−1^) the “ucld.stdev” parameters were all <0.3 with a frequency histogram abutting 0, we chose a strict molecular clock for the analyses [51]. The analyses were performed with two independent chains and 20 million generations and chains were sampled every 1000 generations with a burn-in of 2 million generations.

## Supporting Information

Table S1 Species, location, collection code and Genbank accession numbers of samples (*see Albert et al. 2009 for details)
(0.15 MB DOC)
