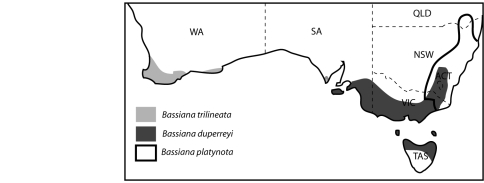# Correction: Evolutionary Diversification of the Lizard Genus *Bassiana* (Scincidae) across Southern Australia

**DOI:** 10.1371/annotation/58397055-6e7b-4588-9176-bb45fb7efd51

**Published:** 2010-10-08

**Authors:** Sylvain Dubey, Richard Shine

Figure 1 was incorrectly formatted. Tasmania should lie outside of mainland Australia. See the correct figure here: 

**Figure:**