# Supplementary material for: Evolutionary Diversification of the Lizard Genus Bassiana (Scincidae) across Southern Australia
Source: PLoS One. 2010 Sep 24;5(9):e12982. doi: 10.1371/journal.pone.0012982 (PMC2945320; doi:10.1371/journal.pone.0012982)
Supplement: Table S1 — Species, location, collection code and Genbank accession numbers of samples (*see Albert et al. 2009 for details) (0.15 MB DOC) [file pone.0012982.s001.doc]

Table S1 Species, location, collection code and Genbank accession numbers of samples (*see Albert et al. 2009 for details)

| **Species** | **Location** | **Number on Fig.2** | **Collection code** | **GenBank Access (ND4/ND2)** |
| --- | --- | --- | --- | --- |
| *Bassiana trilineata* | 1.5k W Mundaring Weir Wall, WA | 1 | ABTC58010 | GU812066/GU811969 |
| *Bassiana trilineata* | Shannon Rock, WA | 2 | ABTC11246 | GU812067/GU811970 |
| *Bassiana trilineata* | 18k W Denmark, WA | 3 | ABTC53636 | GU812071/GU811974 |
| *Bassiana trilineata* | 65k W Esperance, Lort River, WA | 4 | ABTC53635 | GU812070/GU811973 |
| *Bassiana trilineata* | Pink Lake, Esperance, WA | 5 | ABTC53628 | GU812068/GU811971 |
| *Bassiana trilineata* | Pink Lake, Esperance, WA | 5 | ABTC53629 | GU812069/GU811972 |
| *Bassiana trilineata* | Coffin Bay, SA | 6 | ABTC73433 | GU812061/GU811964 |
| *Bassiana trilineata* | Coffin Bay, SA | 6 | ABTC73436 | GU812062/GU811965 |
| *Bassiana trilineata* | Wanna, SA | 7 | ABTC15403 | GU812057/GU811960 |
| *Bassiana trilineata* | Wanna Dunes Lincoln, SA | 7 | ABTC58717 | GU812064/GU811967 |
| *Bassiana trilineata* | Wanna Dunes Lincoln NP, SA | 7 | ABTC58718 | GU812058/GU811961 |
| *Bassiana trilineata* | 22.4k SSE Port Lincoln, SA | 8 | ABTC39561 | GU812063/GU811966 |
| *Bassiana trilineata* | 20k SSE Port Lincoln, SA | 8 | ABTC69267 | GU812060/GU811963 |
| *Bassiana trilineata* | 18k S Port Lincoln, SA | 8 | ABTC69268 | GU812065/GU811968 |
| *Bassiana trilineata* | Port Lincoln, SA | 8 | ABTC73445 | GU812059/GU811962 |
| *Bassiana platynota* | Cooma, NSW | 9 | ABTC40980 | GU812048/GU811951 |
| *Bassiana platynota* | near Barry, NSW | 10 | ABTC11219 | GU812047/GU811950 |
| *Bassiana platynota* | near Barry, NSW | 10 | ABTC11220 | GU812052/GU811955 |
| *Bassiana platynota* | near Barry, NSW | 10 | ABTC11221 | GU812053/GU811956 |
| *Bassiana platynota* | Woodford, NSW | 11 | ABTC06966 | GU812042/GU811945 |
| *Bassiana platynota* | near Ebenezer, NSW | 12 | ABTC11217 | GU812045/GU811948 |
| *Bassiana platynota* | Kanangra Walls, NSW | 13 | ABTC06962 | GU812044/GU811947 |
| *Bassiana platynota* | Dharawal, NSW | 14 | BMC-0001 | GU812050/GU811952 |
| *Bassiana platynota* | Dharawal, NSW | 14 | BMC-0004 | GU812051/GU811954 |
| *Bassiana platynota* | Royal NP, NSW | 15 | DAP-0533 | GU812049/GU811952 |
| *Bassiana platynota* | Monkey Gum, Morton NP, NSW | 16 | DAP-0137 | GU812041/GU811944 |
| *Bassiana platynota* | Monkey Gum, Morton NP, NSW | 16 | DAP-0333 | GU812040/GU811943 |
| *Bassiana platynota* | Monkey Gum, Morton NP, NSW | 16 | DAP-0127 | GU812055/GU811958 |
| *Bassiana platynota* | Monkey Gum, Morton NP, NSW | 16 | DAP-0147 | GU812054/GU811957 |
| *Bassiana platynota* | 13k W Bendemeer, NSW | 17 | ABTC11218 | GU812046/GU811949 |
| *Bassiana platynota* | 35k E Walcha, NSW | 18 | ABTC03999 | GU812043/GU811946 |
| *Bassiana platynota* | New England NP, NSW | 19 | ABTC11222 | GU812056/GU811959 |
| *Bassiana duperreyi* | Mt Murphy, VIC | 20 | Z7615 | GU812000/GU811903 |
| *Bassiana duperreyi* | Gingera, ACT | 21 | T064 | GU811977/GU811880 |
| *Bassiana duperreyi* | Ginini, ACT | 22 | T040 | GU811975/GU811878 |
| *Bassiana duperreyi* | Brindabella RD, NSW | 23 | BrindRD | GU811983/GU811886 |
| *Bassiana duperreyi* | Coree Flat, NSW | 24 | CF1 | GU811979/GU811882 |
| *Bassiana duperreyi* | Coree Flat, NSW | 24 | CF2 | GU811980/GU811883 |
| *Bassiana duperreyi* | Coree Flat, NSW | 24 | CF3 | GU811981/GU811884 |
| *Bassiana duperreyi* | Coree Flat, NSW | 24 | CF4 | GU811982/GU811885 |
| *Bassiana duperreyi* | Coree Flat, NSW | 24 | T055 | GU811978/GU811881 |
| *Bassiana duperreyi* | Collector, NSW | 25 | ABTC11243 | GU811985/GU811888 |
| *Bassiana duperreyi* | Collector, NSW | 25 | ABTC11245 | GU811984/GU811887 |
| *Bassiana duperreyi* | Piccadilly Circus, ACT | 26 | T047 | GU811976/GU811879 |
| *Bassiana duperreyi* | 20.3k N Abercrombie R, NSW | 27 | ABTC57498 | GU811986/GU811889 |
| *Bassiana duperreyi* | 7.5k S Abercrombie R, NSW | 27 | ABTC57507 | GU811987/GU811890 |
| *Bassiana duperreyi* | Kangaroo Island, SA, 360054S 1370051E | 28 | ABTC33362 | GU812001/GU811904 |
| *Bassiana duperreyi* | Kangaroo Island, SA, 360054S 1370051E | 28 | ABTC33397 | GU812007/GU811910 |
| *Bassiana duperreyi* | Kangaroo Island, SA, 355450S 1363613E | 28 | ABTC33431 | GU812002/GU811905 |
| *Bassiana duperreyi* | Kangaroo Island, SA, 355450S 1363613E | 28 | ABTC33437 | GU812003/GU811906 |
| *Bassiana duperreyi* | Kangaroo Island, SA, 360245S 1364255E | 28 | ABTC33452 | GU812004/GU811907 |
| *Bassiana duperreyi* | Kangaroo Island, SA, 355523S 1364301E | 28 | ABTC33466 | GU812005/GU811908 |
| *Bassiana duperreyi* | Kangaroo Island, SA, 355431S 1364336E | 28 | ABTC33467 | GU812006/GU811909 |
| *Bassiana duperreyi* | Kangaroo Island, SA, 354730S 1365530E | 28 | ABTC33500 | GU812015/GU811918 |
| *Bassiana duperreyi* | 8.5k SSE Second Valley, SA | 29 | ABTC33274 | GU812013/GU811916 |
| *Bassiana duperreyi* | 1.7k N Mount Billy, SA | 30 | ABTC94916 | GU812034/GU811937 |
| *Bassiana duperreyi* | 5.6k SE Mt Magnificent, SA | 31 | ABTC95063 | GU812033/GU811936 |
| *Bassiana duperreyi* | Cherry Gardens, SA | 32 | ABTC15848 | GU812010/GU811913 |
| *Bassiana duperreyi* | 3.5k W Gumeracha, SA | 33 | ABTC17525 | GU812011/GU811914 |
| *Bassiana duperreyi* | Totness CP, SA | 34 | ABTC74477 | GU812028/GU811931 |
| *Bassiana duperreyi* | Kaiser Stuhl CP, SA | 35 | ABTC57535 | GU812029/GU811932 |
| *Bassiana duperreyi* | Mt Gawler NFR, SA | 36 | ABTC74256 | GU812031/GU811934 |
| *Bassiana duperreyi* | Devils Gully NFR, SA | 37 | ABTC74248 | GU812023/GU811926 |
| *Bassiana duperreyi* | Scott Creek CP, SA | 38 | ABTC74406 | GU812032/GU811935 |
| *Bassiana duperreyi* | 9k E Mt Compass, SA | 39 | ABTC33246 | GU812012/GU811915 |
| *Bassiana duperreyi* | 2.5k NE Mt Compass, SA | 39 | ABTC33275 | GU812014/GU811917 |
| *Bassiana duperreyi* | Spring Mount CP, SA | 40 | ABTC68913 | GU812027/GU811930 |
| *Bassiana duperreyi* | 2.2k ESE Deep Creek HS, SA | 41 | ABTC58040 | GU812021/GU811924 |
| *Bassiana duperreyi* | 6k SE Meningie, SA | 42 | ABTC34348 | GU812016/GU811919 |
| *Bassiana duperreyi* | 13.5k ENE Salt Creek, SA | 43 | ABTC35073 | GU812017/GU811920 |
| *Bassiana duperreyi* | 3.5k W Lesron HS, SA | 44 | ABTC38069 | GU812035/GU811938 |
| *Bassiana duperreyi* | 35k ENE Kingston SE, SA | 45 | ABTC36241 | GU812025/GU811928 |
| *Bassiana duperreyi* | 3.8k SSW Salt Creek Trig, SA | 45 | ABTC37399 | GU812018/GU811921 |
| *Bassiana duperreyi* | E of Kingston SE, SA | 45 | ABTC58806 | GU812022/GU811925 |
| *Bassiana duperreyi* | Alaman Station, SA | 46 | ABTC53794 | GU812036/GU811939 |
| *Bassiana duperreyi* | 10.4k SSE The Gap, SA | 47 | ABTC37618 | GU812019/GU811922 |
| *Bassiana duperreyi* | 13.5k Frances, SA | 48 | ABTC57701 | GU812020/GU811923 |
| *Bassiana duperreyi* | Penola State Forest, SA | 50 | ABTC14283 | GU812009/GU811912 |
| *Bassiana duperreyi* | Canunda NP, SA | 51 | ABTC14265 | GU812008/GU811911 |
| *Bassiana duperreyi* | 8.4k W Kongorong, SA | 52 | ABTC37728 | GU812037/GU811940 |
| *Bassiana duperreyi* | Cape Banks, SA | 53 | ABTC54773 | GU812024/GU811926 |
| *Bassiana duperreyi* | Piccaninnie Ponds, SA | 54 | ABTC37429 | GU812030/GU811933 |
| *Bassiana duperreyi* | Bucks Lake Game Res, SA | 55 | ABTC54772 | GU812038/GU811941 |
| *Bassiana duperreyi* | 0.7k NE Donovans, SA | 56 | ABTC37423 | GU812039/GU811942 |
| *Bassiana duperreyi* | 18.3k N Coonawarra, SA | 57 | ABTC37683 | GU812026/GU811929 |
| *Bassiana duperreyi* | 5k W Dreeite, VIC | 58 | ABTC04110 | GU811996/GU811899 |
| *Bassiana duperreyi* | Dreeite, VIC | 58 | ABTC11242 | GU811995/GU811898 |
| *Bassiana duperreyi* | Rockbank, VIC | 59 | ABTC11249 | GU811999/GU811902 |
| *Bassiana duperreyi* | Westernport Bay, VIC | 60 | ABTC11247 | GU811997/GU811900 |
| *Bassiana duperreyi* | Westernport Bay, VIC | 60 | ABTC11248 | GU811998/GU811901 |
| *Bassiana duperreyi* | 12k N Triabunna, TAS | 61 | ABTC23542 | GU811988/GU811891 |
| *Bassiana duperreyi* | 8k W Campbell Town, TAS | 62 | ABTC23543 | GU811989/GU811892 |
| *Bassiana duperreyi* | Gladstone, TAS | 63 | R05622 | GU811990/GU811893 |
| *Bassiana duperreyi* | Emita, Flinders Island, TAS | 64 | ABTC23588 | GU811994/GU811897 |
| *Bassiana duperreyi* | Emita, Flinders Island, TAS | 64 | ABTC23592 | GU811993/GU811896 |
| *Bassiana duperreyi* | Flinders Island, TAS | 64 | ABTC23606 | GU811991/GU811894 |
| *Bassiana duperreyi* | Flinders Island, TAS | 64 | ABTC23610 | GU811992/GU811895 |
| *Cordylus warreni* |  |  |  | AB079613* |
| *Eumeces egrerius* |  |  |  | AB016606* |
| *Lacerta viridis* |  |  |  | AM176577* |
| *Lepidophyma flavimaculatum* |  |  |  | AB162908* |
| *Takydromus takydromoides* |  |  |  | AB080237* |
